# Fully automated deep learning-based localization and segmentation of the locus coeruleus in aging and Parkinson’s disease using neuromelanin-sensitive MRI

**DOI:** 10.1007/s11548-021-02528-5

**Published:** 2021-11-19

**Authors:** Max Dünnwald, Philipp Ernst, Emrah Düzel, Klaus Tönnies, Matthew J. Betts, Steffen Oeltze-Jafra

**Affiliations:** 1 Department of Neurology, Faculty of Medicine, Otto von Guericke University Magdeburg (OVGU), Magdeburg, Germany; 2 Faculty of Computer Science, OVGU, Magdeburg, Germany; 3 German Center for Neurodegenerative Diseases (DZNE), Magdeburg, Germany; 4 Institute of Cognitive Neurology and Dementia Research (IKND), Faculty of Medicine, OVGU, Magdeburg, Germany; 5 Institute of Cognitive Neuroscience, University College London, London, Great Britain UK; 6 Center for Behavioral Brain Sciences (CBBS), Magdeburg, Germany

**Keywords:** Localization, Segmentation, Deep learning, Locus coeruleus

## Abstract

****Purpose**:**

Development and performance measurement of a fully automated pipeline that localizes and segments the locus coeruleus in so-called neuromelanin-sensitive magnetic resonance imaging data for the derivation of quantitative biomarkers of neurodegenerative diseases such as Alzheimer’s disease and Parkinson’s disease.

****Methods**:**

We propose a pipeline composed of several 3D-Unet-based convolutional neural networks for iterative multi-scale localization and multi-rater segmentation and non-deep learning-based components for automated biomarker extraction. We trained on the healthy aging cohort and did not carry out any adaption or fine-tuning prior to the application to Parkinson’s disease subjects.

****Results**:**

The localization and segmentation pipeline demonstrated sufficient performance as measured by Euclidean distance (on average around 1.3mm on healthy aging subjects and 2.2mm in Parkinson’s disease subjects) and Dice similarity coefficient (overall around $71\%$ on healthy aging subjects and $60\%$ for subjects with Parkinson’s disease) as well as promising agreement with respect to contrast ratios in terms of intraclass correlation coefficient of $\ge 0.80$ for healthy aging subjects compared to a manual segmentation procedure. Lower values ($\ge 0.48$) for Parkinson’s disease subjects indicate the need for further investigation and tests before the application to clinical samples.

****Conclusion**:**

These promising results suggest the usability of the proposed algorithm for data of healthy aging subjects and pave the way for further investigations using this approach on different clinical datasets to validate its practical usability more conclusively.

## Introduction

The locus coeruleus (LC), a small cylindrical structure in the brainstem of about 2mm in diameter and 12 to 17mm in length [10], is gaining rapidly increasing interest in the neuroscientific community. The LC is one of the earliest structures to be affected in neurodegenerative diseases such as Alzheimer’s disease (AD) [4] and Parkinson’s disease (PD) [5]. Thus, there has been considerable interest to develop magnetic resonance imaging (MRI) techniques to assess the integrity of the LC in vivo. Through utilizing so-called neuromelanin-sensitive MRI techniques, that take advantage of the large amounts of neuromelanin—a pigmented polymer produced from the oxidation of catecholamines including noradrenaline in the LC—it is now possible to investigate the LC in vivo. This may help to further understand the pathogenesis of neurodegenerative diseases [3] and provide novel insights into cognitive and behavioral symptoms necessary to develop effective therapies [24].

A reliable segmentation is often the requirement for a robust extraction of (potential) biomarkers. In the case of in vivo LC segmentation, this poses considerable challenges due to the small size of the structure and the comparatively coarse resolution of MRI acquisitions. An increase in the resolution, however, jeopardizes the already relatively low signal-to-noise ratio (SNR). Although reasonable compromises can be found [2], the issue remains challenging. This is reflected by low inter-rater agreement between expert raters, which ranges between a Dice similarity coefficient (DSC) of 0.499 [1], 0.64 [25] and 0.68 (ours). However, properties of the measured hyperintense regions on so-called neuromelanin-sensitive MR images have been shown to correspond to LC properties determined in post-mortem studies, such as general position and dimensions, as well as LC cell density [15] and age-related effects of neuromelanin aggregation [2].

Reliable methods for automated LC segmentation and extraction of potential, imaging-based LC biomarkers are highly desirable to improve objectivity and comparability between studies and facilitate the ongoing search for early, in vivo imaging-based biomarkers for neurodegenerative diseases. This work offers four contributions to this field. First, we propose a novel iterative, multi-scale strategy for the LC localization network, which is significantly more precise, requires less graphics processing unit (GPU) memory and less training time than our previously published method [8]. Secondly, we investigate the usage and advantages of the availability of multiple raters. We propose two different approaches that make use of multiple manually segmented LC masks for the training of the segmenter network and found improved performance compared to single rater training. Third, we apply our pipeline trained on a healthy aging dataset to unseen data of PD subjects without any fine-tuning and evaluate its performance. Finally, we enable fully automated LC analysis based on the most commonly extracted potential biomarker, contrast ratios (CRs) [17], by segmenting the pons using a 3D-Unet [6, 9] and applying several robust post-processing steps.

## Related work

The vast majority of publications analyzing the LC rely on purely manual methods [17]. However, very few (semi-) automatic approaches have been proposed which mostly build on atlas registration [15, 27] or dynamic atlas-based methods yielding moderate performance (DSC of $0.45\pm 0.25$ [21] and 0.4 [1]). Furthermore, several more classical methods have been applied to the task: region growing [20], clustering with Gaussian mixture models and anatomical prior knowledge [18], intensity and shape models [26], active contours [22] as well as a variety of essentially intensity thresholding-based approaches [7, 12].

To the best of our knowledge, our previous work that explored the use of a 3D-Unet [6] variant for this task [9] as well as a preceding coordinate regression-based localization [8] are the only publications to date to investigate deep learning-based methods in segmenting the LC. However, for the Substantia Nigra, which can be visualized using the same MRI acquisitions, there are a few publications that make use of deep learning-based approaches, most notably [16] and [23]. In fact, our approach shares several similarities to [16], but we deviate for instance in using a different localization approach and employing multi-rater trainings for the segmentation.

**Fig. 1:**
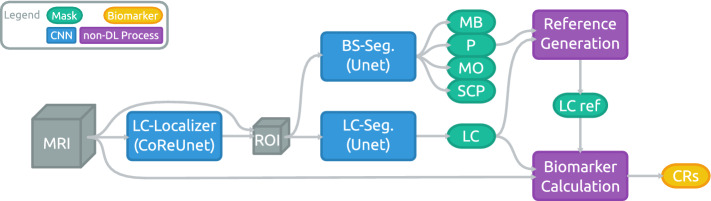
Schematic illustration of the proposed approach for an automated LC analysis pipeline. The output of the brainstem substructure (BS) segmentation network are masks for midbrain (MB), pons (P), medulla oblongata (MO) and superior cerebellar peduncle (SCP)

## Methods and materials

This section describes our proposed pipeline. As visualized in Fig. 1, a preceding localization of the LC is used to extract a Region of Interest (ROI), which is then processed by networks to segment the LC itself as well as several brainstem substructures. The latter include the pons, which is needed to derive the reference regions required for the extraction of CRs.

### Datasets

We used two different datasets: one dataset comprising healthy aging subjects [2, 8], which was used for the development, training and evaluation of the method as well as one comprising PD subjects, that was used merely for evaluation. The healthy aging dataset (HAD) comprises data of 82 subjects, of which 25 are younger (22–30 years old; 13 male, 12 female) and 57 are older healthy adults (61–80 years; 19 male, 38 female). The LCs were manually segmented by two expert raters (R1, R2). The Parkinson’s disease dataset (PDD) contains data of 22 subjects (10 male, 12 female) that have been diagnosed with PD according to UK Parkinson’s Disease Brain Bank criteria [13]. Their age ranges between 48 and 77 years and is 65.55 on average. The LCs were segmented by just one of the raters (R1).

HAD and PDD were acquired using the same protocol, i.e., applying T$_{1}$-weighted FLASH 3T MRI whole-brain acquisitions with an isotropic resolution of 0.75mm$^3$. Prior to delineation, the data was upsampled to 0.375mm$^3$ by means of a sinc filter. Additionally, a bias field correction was applied. The final step of the pre-processing was normalization of the data, such that the intensity values of each acquisition had a mean of 0 and a standard deviation of 1.

### Iterative multi-scale localization of the LC

As described in previous work [8], we use a preceding localization network, which regresses the center of mass of the LC. This allows focusing just on the nucleus itself and its vicinity for the following processes and reduces the amount of false positives. The localization architecture, CoRe-Unet, combines a 3D-Unet and a Differentiable Spatial to Numerical Transform (DSNT) layer [19], which enables the direct regression of coordinates utilizing the Unet’s output, a heatmap, that gives insights into the network’s behavior. The Euclidean distance was used as the loss function. Augmentations in the form of mild random affine transformations have been applied as they have shown to yield preferable performance in earlier work. We investigated two different approaches for the training and application of this network: In the first approach, and in accordance with prior work, we used the acquisitions in their original isotropic resolution of $0.75\mathrm {mm}^3$ as a whole for training and inference, which dictated the upper limit of the batch size (2) due to GPU memory limitations. We refer to this version simply as *CoRe-Unet*. In the second approach, we investigated a new iterative multi-scale approach, that exposes the network to patches (64$^3$ voxels) of different resolutions of the data during training: 0.375mm$^3$, 0.75mm$^3$, 1.5mm$^3$ and 3mm$^3$. In the inference case, the network is applied four times consecutively to patches of the input volume that are interpolated to increasing resolutions with increasing iteration. In this process, the network’s prediction of the LC position in the current iteration is used to extract the patch of the next stage (with higher resolution). Due to the smaller patches that can be used, training time and required GPU memory were drastically reduced, allowing a batch size of 32 and an increased number of feature maps in the network, making it equivalent to the segmenter architectures. We refer to this version as *MS-CoRe-Unet*.

### Multi-rater segmentation of the LC

The segmentation networks in the proposed pipeline are all based on a slightly adapted version of 3D-Unet [6, 9]. They were trained with a fuzzy DSC loss function in a patch-based manner ($64^3$ voxels) and a batch size of 32. Apart from a random translation, no further augmentation techniques were applied to avoid the need for interpolation of both, the target masks and the already weak imaging signal. As a post-processing step to address remaining false positive regions, we performed a connected component analysis and kept only the largest connected component as the final prediction.

As the training target for LC segmentation, we used manual masks created by two expert raters. Besides training a network with each of them (resulting in *NetR1* and *NetR2*), we explored two ways of combining them and investigating possible improvements in terms of objectiveness of the segmentation results. Hence, we trained a network with the intersection of the two raters’ masks (*NetInt*) and another with randomly switching between both raters during the training (*NetRnd*).

**Fig. 2:**
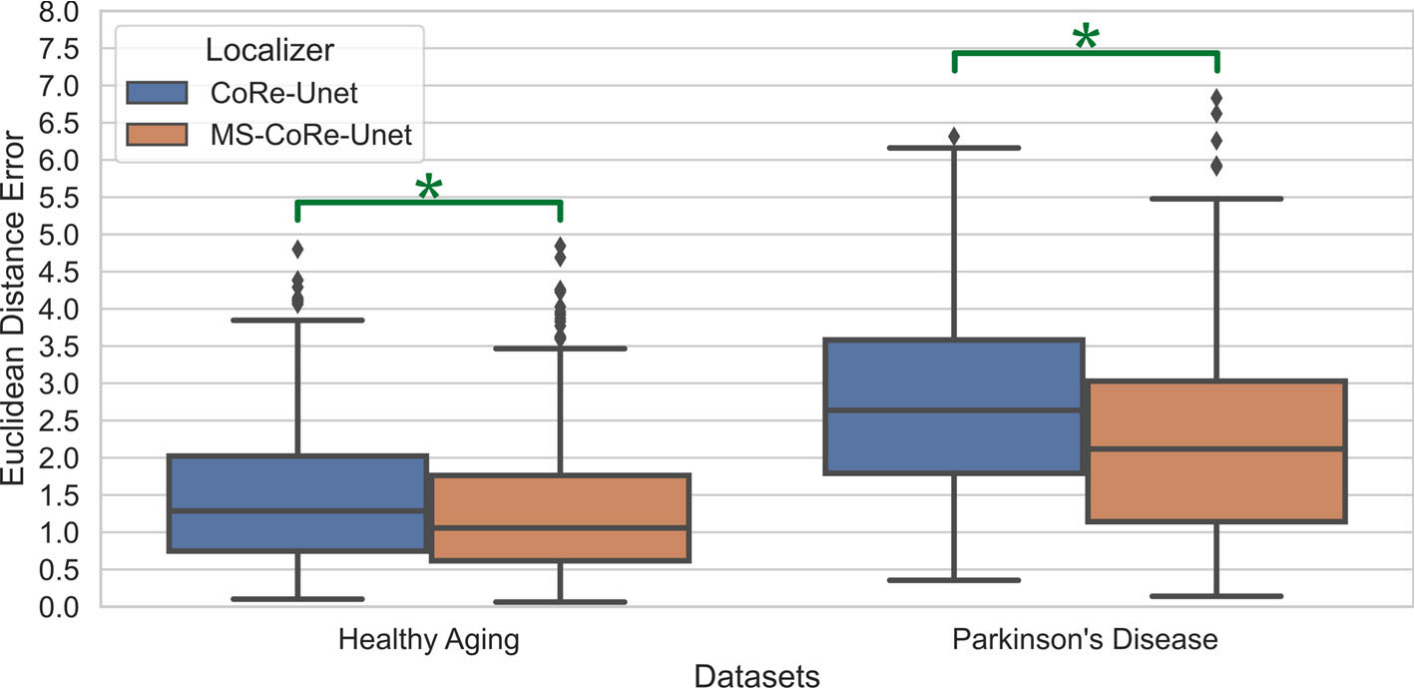
Boxplots of Euclidean distance errors measured in millimeter. MS-CoRe-Unet resulted in statistically significantly smaller errors on both, HAD (*p* value = $1.16\mathrm {e}-11$) and PDD (*p* value = $6.17\mathrm {e}-6$). Three outliers on the PDD have been omitted for better visualization

### Fully automated LC analysis

To complete the goal of fully automated CR-based analysis [2, 17], we require the reference regions that are used to calculate the median or maximum LC intensity ratio with respect to the median/maximum intensity of the surroundings and are typically positioned in both hemispheres of the pons to account for lateral bias effects [2]. Previously, they were generated semi-automatically [2] by manually placing a fixed-sized cuboid in a template which was then propagated to each sample space requiring several registration operations. We propose a faster, fully automated approach by training a 3D-Unet analogously to the LC segmenter to create pons masks and then, apply several robust post-processing steps to obtain valid reference regions. To this end, the pons masks are split in a Voronoi diagram fashion. That is every pons voxel is assigned to the LC center point that has the shortest distance to it, thus creating a separation layer which is defined by the previously determined LC centers. At the position of the respective center points of the resulting pons halves, a fixed-sized cuboid ($20^3$ voxels) is placed.

As a target for the pons segmentation, we used the results of the Brainstem Substructure functionality [14] in FreeSurfer [11] (version 7.1.0), which can provide masks for midbrain (MB), pons (P), medulla oblongata (MO) and superior cerebellar peduncle (SCP). For its application, however, the resolution of the data had to be reduced by means of sinc interpolation to a third, i.e., $1.125\mathrm {mm}^3$ isotropic resolution. Thus, the obtained substructure masks showed slight interpolation artifacts after upscaling them to be used as a target with the same resolution as the other masks. Furthermore, one of the samples had to be excluded for the pons segmentation training because the Brainstem Substructure tool failed to generate an output mask. Although our network is able to segment all four subregions, we solely utilized the pons mask in this work. Using a neural network instead of the FreeSurfer pipeline has the principle advantage of faster processing since an inference of the network requires merely seconds while the application of the FreeSurfer pipeline typically requires several hours.

### Evaluation scheme and metrics

To minimize the chance of a possible bias in both validation and test sets as well as to assess the effect of set composition (combinations of subjects in the sets), we used a nested cross-validation scheme with three outer folds and five inner folds for evaluation. Hence, the data was randomly split into three subsets and for three iterations. One of them was used as the test set, while the other two are once more randomly split into five subsets. On each of these, another fivefold cross-validation was performed to yield validation (one of the subsets) and training sets (the remaining four subsets). In the resulting 15 combinations, we used the respective validation loss for early stopping and calculated the performances of the five versions of the inner cross-validation on their respective test set. We maintained the same ratio of younger to older subjects in every subset throughout the process.

**Table 1:** Means (bold) and standard deviations of DSC (and MRDSC) agreement of the differently trained segmenters on the two datasets. Multi-rater comparison was possible on the HAD only. The inter-rater agreement between R1 and R2 on HAD is $67.54\pm 10.03$%

Net	Healthy aging dataset				PD dataset
	R1	R2	Intersection	MRDSC	R1
NetR1	$\mathbf {70.53\%} \pm 11.12\%$	$\mathbf {69.27\%} \pm 11.19\%$	$\mathbf {69.14\%} \pm 11.94\%$	$\mathbf {69.87\%} \pm 9.84\%$	$\mathbf {57.89\%} \pm 12.90\%$
NetR2	$\mathbf {68.82\%} \pm 11.32\%$	$\mathbf {71.49\%} \pm 11.79\%$	$\mathbf {70.77\%} \pm 11.56\%$	$\mathbf {70.04\%} \pm 10.33\%$	$\mathbf {59.16\%} \pm 12.98\%$
NetInt	$\mathbf {66.06\%} \pm 13.45\%$	$\mathbf {68.41\%} \pm 13.67\%$	$\mathbf {73.92\%} \pm 11.72\%$	$\mathbf {66.81\%} \pm 12.63\%$	$\mathbf {54.13\%} \pm 15.28\%$
NetRnd	$\mathbf {71.59\%} \pm 10.08\%$	$\mathbf {71.83\%} \pm 10.51\%$	$\mathbf {70.77\%} \pm 11.02\%$	$\mathbf {71.71\%} \pm 8.81\%$	$\mathbf {60.87\%} \pm 12.83\%$

We quantified the localization error using the Euclidean distance (measured in millimeter). As for the agreement of the segmentations, we determine the DSC and false discovery rate (FDR) to individual raters as well as their intersection and propose a multi-rater Dice similarity coefficient (MRDSC) to assess the agreement to the set of all raters jointly. It is defined as follows:

$$\begin{aligned} \mathrm {MRDSC}_P(R_1,...,R_n)=\frac{2\sum _{i=1}^n|P\cap R_i|}{n|P|+\sum _{i=1}^n|R_i|} \end{aligned}$$

with *P* the predicted set of LC voxels of the network and $R_i$ the *i*th rater’s selection. We evaluated the similarity of extracted CRs with intraclass correlation coefficients (ICCs). We assessed the statistical significance of all differences we mention using the Welch t test, but report the p value only for certain cases for the sake of clarity.

## Results

We report the results from the previously described evaluation procedure. Although the vast majority of the folds of the nested cross-validations showed no significant differences in performance between them, we found slight deviations in a few cases, indicating that the method and evaluation could profit from an increased dataset size to more conclusively cover the variance of this challenging task.

*Localizer.* As can be seen in Fig. 2, the localization error on the HAD is $1.470\pm 0.877$mm for CoRe-Unet and $1.284\pm 0.838$mm for its iterative multi-scale alternative, MS-CoRe-Unet, which was found to be statistically significantly better than for CoRe-Unet. Larger errors were found for both methods on the PDD with $2.733\pm 1.312$mm (CoRe-Unet) and $2.224\pm 1.425$mm (MS-CoRe-Unet), but the significant distance in performance between the approaches persisted. Most of the error in both cases stems from deviations along the axial axis, i.e., the axis along the rostrocaudal extent of the LC. The dimension-wise average absolute errors in-plane of MS-CoRe-Unet range between 0.238 and 0.395mm, while in the axial direction, 1.138mm and 1.156mm for left and right LC were measured on HAD, but the same effect can be seen on PDD as well. The precision was in all cases of both datasets high enough for the resulting patch ($128^3$) to include the LC masks of both raters entirely.

*Multi-rater Segmentation.* Table 1 shows statistics of the performance obtained with the different segmentation networks on the two datasets. The HAD allows comparisons with the different raters as well as their intersection and the calculation of the MRDSC, that expresses the agreement to both raters jointly. On HAD, the nested cross-validation showed most of the networks to be in or above the range of the inter-rater agreement ($67.54\pm 10.03$%). A notable exception is NetInt, for which lower DSCs were obtained. However, NetInt also produces significantly lower FDRs, which were on average consistently at least 10% less than those of NetR1, NetR2 and NetRnd, regardless of which mask was used as a target (R1 or R2). NetRnd performs statistically significantly better than NetR1 on R2, the intersection, MRDSC and even R1 itself. It outperforms NetR2 on R1 as well as the MRDSC. Furthermore, NetRnd performs better than all other nets on the PDD as well. Nonetheless, all networks show lower agreements on the unseen data of PD subjects.

**Table 2:** Agreement of CRs (in terms of ICCs) from automated LC masks and automatic/semi-automatic reference regions to manual LC masks and semi-automated reference regions. The reported ICCs refer to CRs with median of left LC, median of right LC, maximum of left LC and maximum of right LC, in this order

Reference	Healthy aging dataset	PD dataset
Automatic	0.86, 0.85, 0.80, 0.90	0.50, 0.81, 0.48, 0.63
Semi-automatic	0.91, 0.91, 0.94, 0.99	0.81, 0.94, 0.85, 0.93

*Fully Automated Pipeline for CR Analysis.* The ICCs reported in Table 2 suggest strong agreement of the method on both datasets when semi-automated reference regions are used. For HAD, good agreement was found for the automated reference regions too, while lower values were determined on the PDD.

**Fig. 3:**
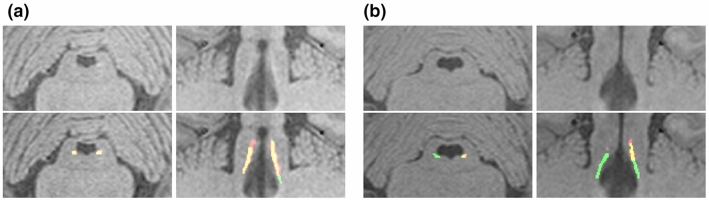
Axial and coronal views of qualitative examples of NetRnd results on PDD. Yellow: agreement with R1, green: false negative region, red: false positive region. a) shows a representative example (left LC: $60.87\%$, right LC: $60.33\%$ (DSC with R1)) and b) shows one of the worst results on a low intensity sample (left LC: $31.93\%$, right LC: $0\%$ (DSC with R1))

## Discussion

The iterative multi-scale approach toward LC localization outperformed our previously published method [8] significantly on both datasets in terms of Euclidean distance error. It furthermore has the advantages of requiring less time for training and lower GPU memory consumption. As observed in [8], the Euclidean distance errors of around 1.3mm on HAD and 2.2mm on PDD are mostly caused by deviations along the axial axis. Since this roughly corresponds to the rostrocaudal axis of the cylindrical LC, which typically has a length between 12 and 17mm, the performance is better than the Euclidean distance might indicate. The precision is therefore more than sufficient for extracting a 128$^3$ voxel patch that contains the entire structure and for consecutively performing the segmentation.

The multi-rater trainings showed several interesting effects. Mostly, the LC segmentation performance was on the same level as the inter-rater agreement, which is arguably the sensible upper limit as further agreement with a single rater would suggest overfitting to this particular rater’s delineation style. That is why it is important to analyze the results wrt. multiple raters. Although NetInt showed comparatively low agreement to both individual raters, the high agreement with their intersection and the concomitantly significantly lower FDR might indicate a more reserved segmentation style, that appears to focus on the more certain regions. NetRnd on the other hand seems to have found a common ground and shows good agreement with both raters, which is especially indicated by the comparison based on MRDSC. It even obtained better agreement with R1 than NetR1, showing that increased rating variance may be beneficial during the training. It might have achieved improved objectivity, since it performed better on the unseen data as well.

When using semi-automatically determined reference regions, our method is able to obtain CRs that show strong agreement with the manually determined ones on both, the HAD and the unseen PDD, suggesting practical usability in this case. The automated reference regions may be used on cohorts with subjects of healthy aging, but caused reduced agreement on PD subjects. The influence of the specific positioning of the cuboid reference region within the pons halves on the respective CRs is an effect that should be quantified in future work. Repeated slight repositioning could yield a variance of considerable magnitude of the feature that could be incorporated in further analyses.

Figure 3 depicts a representative as well as the worst example of the results of NetRnd on PDD. Qualitative assessment suggests that the drop of performance as measured in DSC on PDD is potentially caused by two aspects. First, the presence of motion artifacts that infers with the weak LC signal considerably and second, increased variance indicated for instance by lower LC signal intensity (see Fig. 3b) likely attributed to pathology. Means of adaption to this variance need to be explored to obtain higher and more consistent segmentation performance.

## Conclusion

In this work, we advanced our existing deep learning-based framework [8] in several ways. An iterative multi-scale approach to the LC localization achieved higher precision. Furthermore, we investigated the use of multiple raters masks and found our approach of random switching between them during training to perform best and more objectively. By deriving reference regions from pons masks, we could fully automatize the extraction of the most popular (potential) MRI biomarker, CRs, for assessment of LC structure in vivo. Besides the advantage of being substantially faster, since the inference of a network takes merely seconds, thorough investigation of the results showed good agreement on both healthy aging as well as unseen PD cohorts with semi-automatically generated reference regions and the values derived from the manual procedure indicating high potential for future clinical applicability. The fully automated CR extraction on the other hand requires further analyses and adaption to variance introduced by different subject cohorts in order to pave the way for clinical applicability.

Future work should focus on several open questions. A direct comparison to the few concurrent methods and atlases should be carried out. To further validate the usability of the approach in practical scenarios, a comprehensive evaluation on more datasets should be considered such that not only the effects of more and different subjects cohorts, e.g., with different neurodegenerative pathologies, but also of MRI scanner properties, scanning protocols and motion artifacts on the performance can be assessed and addressed. Finding means of adaption to unseen cohort variance may also enable the exploration of new, more sophisticated potential biomarkers, such as volume [17] or regional intensity gradients [2].

## Data Availability

The data are not publicly available.

## Declarations

### Code availability

The code is currently not publicly available. Publication is planned in the foreseeable future.
